# Exploring Alcohol Withdrawal Syndrome

**Published:** 1997

**Authors:** Deborah A. Finn, John C. Crabbe

**Affiliations:** Deborah A. Finn, Ph.D., is an assistant professor in the Department of Behavioral Neuroscience, Oregon Health Sciences University, and a research pharmacologist at the Dept. of Veterans Affairs Medical Center, Portland, Oregon. John C. Crabbe, Ph.D., is a professor in the Department of Behavioral Neuroscience, Oregon Health Sciences University, director of the Portland Alcohol Research Center, and a research career scientist at the Dept. of Veterans Affairs Medical Center, Portland, Oregon

**Keywords:** AOD withdrawal syndrome, chronic AODE (alcohol and other drug effects), symptom, biological inhibition, neurotransmission, neurochemistry, neurotransmitters, neurotransmitter receptors, animal model, animal strains, membrane channel, genetic mapping, biochemical mechanism, literature review

## Abstract

Alcohol withdrawal syndrome is characterized by hyperactivity of the nervous system. This hyperactivity represents the brain’s attempt to function normally despite the inhibitory effect of chronic alcohol consumption. The syndrome manifests when alcohol consumption ceases. Experimental, clinical, and genetic research have linked the development of withdrawal to alterations in the sensitivity of neuronal communication systems. Early treatment of the syndrome is advised, because the symptom severity may increase with each subsequent episode.

Alcohol withdrawal syndrome consists of unpleasant physical and mental symptoms following the cessation of alcohol consumption. These symptoms may range from mild tremor to hallucinations and convulsions. Repeated withdrawal episodes may contribute to the development of alcohol dependence and to the negative health consequences of drinking. An increased understanding of the genetic and biochemical correlates of the syndrome may lead to improved treatments for withdrawal as well as for other aspects of alcohol dependence.

The symptoms of withdrawal represent a phase in the alcohol-induced cycle of neuronal inhibition and excitation. Research has demonstrated that short-term (i.e., acute) administration of alcohol can alter the release of chemical messengers (i.e., neurotransmitters) from neurons and can disrupt the function of proteins in neuronal membranes, including receptor proteins, which bind to neurotransmitters, and ion channels through which ions^1^ (e.g., sodium or calcium) enter the cell. Following repeated administration of alcohol, the brain attempts to restore normal functioning through adaptations (i.e., tolerance) that reduce alcohol’s initial perturbing effects (figure 1). Long-term exposure to alcohol can lead to physical dependence, in which neuronal adaptations to alcohol become sufficiently pronounced that the brain requires the continued presence of alcohol to function normally. When a person terminates a prolonged drinking session, the adaptations that developed to offset alcohol’s initial inhibitory actions are unopposed, resulting in a rebound hyperexcitability, or withdrawal syndrome.

This article describes the symptoms of alcohol withdrawal; the use of animal models to study the mechanisms and genetics of withdrawal; and the involvement of neurotransmitters, receptors, and ion channels in the syndrome. For a discussion on the role of withdrawal in the development of addiction, see the article by Roberts and Koob, pp. 101–106.

## Symptoms of Alcohol Withdrawal

Alcohol withdrawal is characterized by symptoms of hyperactivity of the autonomic nervous system, a division of the nervous system that helps manage the body’s response to stress (Schuckit et al. 1995; Yost 1996). Alcoholics often continue to consume alcohol to relieve or avoid withdrawal symptoms.

The initial symptoms usually are relatively mild and include anxiety, insomnia, and tremors. These symptoms may begin within 3 to 6 hours following cessation of drinking and often before the blood alcohol concentration (BAC) has returned to zero. The symptoms usually abate within 1 to 3 days. Severe convulsions also may occur within the first 2 days of abstinence in 5 to 10 percent of patients (Victor and Adams 1953; Schuckit et al. 1995; Yost 1996).

**Figure 2:**
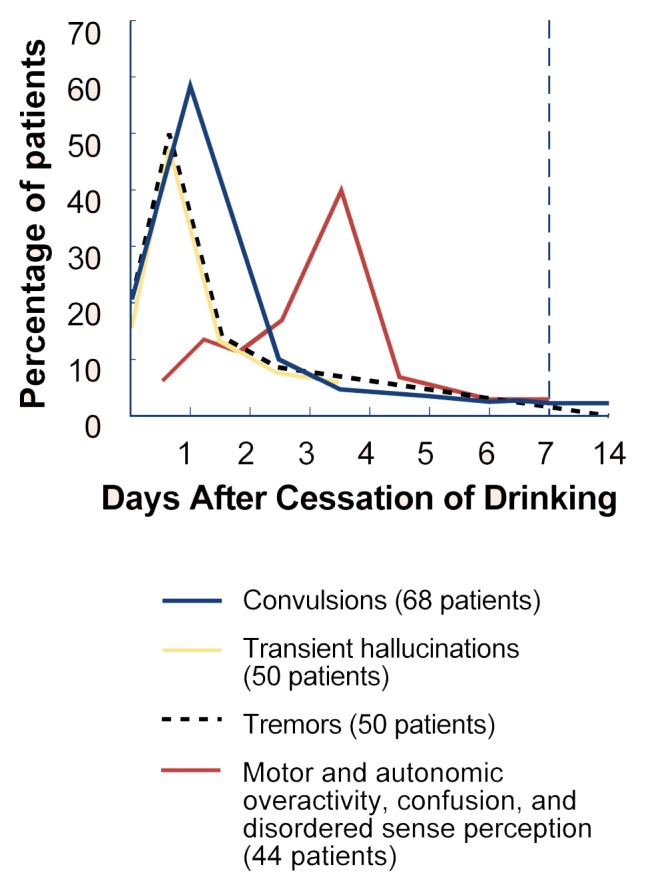
The relation of withdrawal symptoms to cessation of drinking. SOURCE: Adapted from Victor and Adams 1953.

According to clinical studies, approximately 10 percent of alcoholics may develop an additional set of withdrawal symptoms. These symptoms represent a combination of marked autonomic nervous system hyperactivity, including large increases in blood pressure, pulse, breathing rate, and heart rate, as well as elevated body temperature. Drenching sweats can cause severe dehydration, and tremor can become sufficiently pronounced to prevent the patient from performing such simple activities as manipulating an eating utensil. Typically, these more severe symptoms peak in intensity between the third and fourth days of abstinence and generally improve by the fifth day (Schuckit et al. 1995) (figure 2).

Severe alcohol withdrawal is frequently complicated in its later stages by the presence of delirium tremens (DT). Patients with DT are agitated, confused, and disoriented, with delusions and vivid hallucinations. DT may persist until 96 hours after cessation of drinking (Victor and Adams 1953).

## The Kindling Model of Alcohol Withdrawal Severity

Several factors are associated with increased withdrawal severity, including increased alcohol consumption, a greater number of prior withdrawal episodes, the occurrence of medical problems, and the use of other drugs in addition to alcohol (Schuckit et al. 1995). According to one hypothesis (Ballenger and Post 1978), repeated withdrawal episodes increase the severity of subsequent withdrawal syndromes as a result of “kindling.” In kindling, a convulsion-promoting drug or electric shock administered repeatedly at doses below the effective level for a single administration eventually elicits a convulsion after a single administration. Thus, the neurochemical changes that occur during alcohol withdrawal (i.e., reduced function of inhibitory neurotransmission and increased activity of excitatory neurotransmission) not only contribute to the withdrawal syndrome, but also may cause long-term changes in brain excitability by a kindlinglike process (Glue and Nutt 1990). Consistent with the kindling hypothesis, the severity of withdrawal in mice, measured by handling-induced convulsions (HIC’s), increases with repeated exposure to alcohol vapor and subsequent withdrawal episodes compared with mice that received an equivalent dose of alcohol vapor in a single administration (Becker and Hale 1993). Therefore, both animal and human studies indicate a greater likelihood of severe withdrawal symptoms in patients who have experienced a greater number of past withdrawal episodes. These data suggest that early treatment for alcohol withdrawal convulsions may minimize increased withdrawal severity in subsequent episodes by preventing this kindling-like process. Research is needed to confirm this possibility.

## Animal Models of Alcohol Withdrawal

Researchers have used various methods to induce physical dependence in animals in order to define and quantify the withdrawal syndrome. The most common paradigms utilize a nutritionally balanced liquid diet containing alcohol, inhalation of alcohol vapor, or multiple daily doses of alcohol administered either by injection or by a tube inserted into the esophagus (i.e., intubation). Although each procedure has both advantages and disadvantages, each one attempts to achieve relatively stable BAC’s over time so that alcohol dose and duration of exposure can be determined.

Symptoms of alcohol withdrawal in animals include tremors and other motor dysfunction as well as autonomic overactivity (reviewed in Friedman 1980). One of the most commonly studied symptoms is convulsions. Depending on the severity of the withdrawal, convulsions can be spontaneous or induced by handling. Susceptibility to chemically induced convulsions and audiogenic seizures (i.e., seizures elicited by sound stimuli) is also increased during alcohol withdrawal. Additional measures of withdrawal include decreased body temperature (i.e., hypothermia), decreased locomotor activity, increased anxiety, and increased change in behavioral reactivity to stimuli.

Since Goldstein (1973) initially demonstrated that genetic factors can influence withdrawal severity, several genetic animal models—including inbred strains, recombinant inbred strains, and selectively bred lines—have been used to study alcohol withdrawal (for reviews, see Deitrich and Spuhler 1984; Kosobud and Crabbe 1995; Metten and Crabbe 1996). The following subsections focus on the usefulness of genetic animal models and provide an overview of the main findings with each animal model.

### Inbred Strains

An inbred strain is derived from 20 generations of brother-sister matings, resulting in a population of animals that is essentially genetically identical. Thus, trait variation within an inbred strain can be attributed to environmental causes, whereas variation between inbred strains can be attributed to genetic causes. Studies documenting differences in alcohol withdrawal severity among inbred strains show clear evidence of genetic influence (Kakihana 1979; Crabbe et al. 1983).

One advantage of testing inbred strains is that genetic correlations among alcohol-related traits can be estimated. Using 20 inbred mouse strains, Crabbe and colleagues (1983) correlated ratings of alcohol withdrawal severity with 13 different measures of alcohol sensitivity and found no strong genetic correlations between withdrawal severity and other measures of alcohol sensitivity. Recently, the magnitude of acute withdrawal severity in inbred animal strains following a single injection of alcohol was found to be similar to that resulting from an injection of the barbiturate pentobarbital. That is, animals with high pentobarbital withdrawal also exhibited high withdrawal to alcohol. This finding suggests that some genes may influence withdrawal severity from multiple depressant drugs.

### Selective Breeding

Another method for testing the role of genes in the expression of withdrawal severity and its relation to other traits utilizes selective breeding. For this purpose, researchers often use bidirectional selection, in which one line is bred for maximal expression of a given trait and the other is bred for minimal expression of that trait. Bidirectional selection for many generations produces animals that differ dramatically in the selection trait and also differ with respect to the genes relevant to the selection trait. This strategy has been used to generate lines of mice that differ in withdrawal severity following chronic alcohol administration. Withdrawal seizure-prone (WSP) and -resistant (WSR) mice were generated by breeding for increased and decreased HIC severity, respectively, after withdrawal from 72 hours of exposure to alcohol vapor. Under these conditions, WSP mice exhibited a 10-fold increase in withdrawal severity compared to WSR mice.

During the past 15 years, the WSP and WSR lines have been tested using various behavioral tasks and with various drugs (Kosobud and Crabbe 1995; Metten and Crabbe 1996). Alcohol withdrawal tremors, in addition to HIC, are more severe in WSP mice compared with WSR mice. However, the lines do not appear to differ with respect to other indicators of withdrawal severity, such as decreased activity. Acute withdrawal severity following a single administration of an anesthetic dose of alcohol was also significantly greater in WSP mice compared with WSR mice.

Consistent with results using inbred strains, WSP and WSR mice not previously exposed to alcohol did not differ from each other in sensitivity to several other effects of alcohol.

## Quantitative Trait Locus Analysis

An additional strategy for understanding mechanisms contributing to genetic differences in alcohol withdrawal severity involves the identification of the specific genes that confer sensitivity or resistance to a particular drug-related behavior. Quantitative traits (e.g., withdrawal severity) are continuously distributed in a population—that is, individual people or animals vary in the degree to which they exhibit such traits. A quantitative trait locus (QTL) is a small section of chromosomal DNA that has been demonstrated to contain a gene or genes that influence a specific trait. Because of the high degree of similarity between locations of specific genes on mouse and human chromosomes (Copeland et al. 1993), when a QTL is mapped in mice, it is highly likely that the human chromosomal map site can be specified.

A recent QTL analysis of withdrawal severity from a single injection of alcohol (i.e., acute withdrawal) has been successfully completed in three distinct genetic populations (Buck et al. 1997). Among other results, a QTL on chromosome 11 was mapped to a location near a cluster of genes that affect the synthesis of a receptor involved in alcohol’s effects (i.e., the gamma-aminobutyric acid [GABA_A_] receptor). Studies are under way to link the function of specific genes to acute alcohol withdrawal severity (Buck et al. 1997).

## Neurotransmitters and Neuromodulators

**Figure 3:**
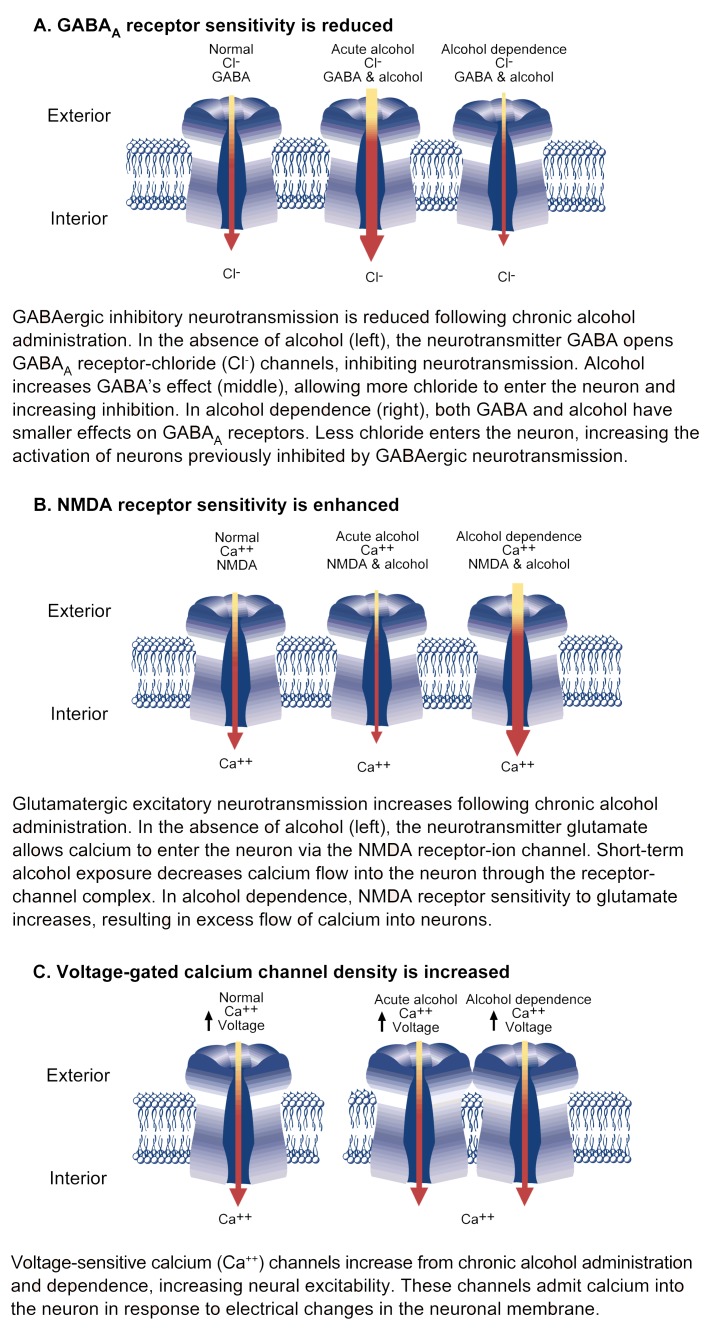
Ion channel adaptations revealed by alcohol withdrawal. Changes in ion channel sensitivity or number during alcohol withdrawal results in decreased inhibitory and increased excitatory receptor function.

The following subsections briefly summarize the effects of chronic alcohol exposure on neurotransmitter receptors and ion channels (figure 3).

### GABA_A_ Receptor Complex

The amino acid GABA is the principal inhibitory neurotransmitter in the brain. GABA functions by attaching to a binding site on GABA_A_ receptors, thereby causing a pore in the cell membrane to open and admit chloride ions.^2^ The flow of these negatively charged ions into the neuron renders it less sensitive to further neurotransmission. GABA_A_ receptors—with binding sites for various molecules and the chloride ion channel—form integrated complexes of five protein subunits (Morrow 1995; Buck 1996).

Various substances that bind to GABA receptors (i.e., GABAergic ligands) can reduce alcohol withdrawal, including benzodiazepine sedatives (e.g., Valium^®^) and certain steroids. The anticonvulsant effect of these substances is shown by reductions in both HIC and sensitivity to chemically induced convulsions. Depending on the ligand, experiments have demonstrated both tolerance and sensitization to the anticonvulsant effects of GABAergic ligands during withdrawal.

Although alcohol’s initial effects are inhibitory, chronic alcohol administration decreases the ability of GABA, benzodiazepines, and barbiturates to activate the inhibitory effect of GABA_A_ receptors in alcohol-dependent animals (i.e., tolerance) (reviewed in Morrow 1995; Buck 1996). In addition, chronic alcohol administration decreases chloride uptake induced by the GABA_A_ activator (i.e., agonist) muscimol and reduces the augmenting effect of benzodiazepines and barbiturates on muscimol-stimulated chloride ion uptake. After alcohol withdrawal, the reduced effectiveness of benzodiazepines on agonist-stimulated chloride uptake is reversed.

Studies in mice, rats, and neuronal cell cultures show that chronic alcohol exposure alters the activity of genes that direct the synthesis of GABA_A_ receptor component subunits, increasing the levels of some while decreasing others. In all studies, chronic alcohol administration decreased levels of the subunit termed α_1_. This subunit also was found to be decreased in alcohol-dependent WSP mice, but not in WSR mice, suggesting the possibility of a genetic link between alcohol withdrawal severity and decreased α_1_ subunit level. Overall, changes in the level of GABA_A_ receptor subunits following chronic alcohol administration may reflect an alteration in the assembly of GABA_A_ receptors, which, in turn, may underlie some of the cellular and behavioral adaptations in alcohol dependence and withdrawal.

### Steroidal GABA Agonists

Allopregnanolone—a product of the metabolism of the hormone progesterone—is the most potent steroidal modulator of GABA_A_ receptor activity. Found in the brain and in the bloodstream, allopregnanolone is thought to be synthesized by the adrenal glands and, in females, by the ovaries; some synthesis also may occur in the brain. Allopregnanolone binds to GABA_A_ receptors at a site distinct from the binding sites for benzodiazepines, barbiturates, and GABA. As a GABA agonist, allopregnanolone’s overall effects are inhibitory, and its binding to GABA_A_ receptors suppresses the development of convulsions. Chronic alcohol administration produces an increased responsiveness (i.e., sensitization) to the anticonvulsant effect of allopregnanolone. In addition, the allopregnanolone-induced augmentation of GABA-stimulated chloride ion uptake increased by 42 percent in alcohol-withdrawing rats compared with rats not administered alcohol (Devaud et al. 1996). Similar results were found with another potent steroidal GABA_A_ agonist, tetrahydrodeoxycorticosterone, indicating that the sensitization of GABA_A_ receptors to neuroactive steroids during alcohol withdrawal is not unique to allopregnanolone. This enhancement of steroidal agonist action during alcohol withdrawal contrasts with the reduced effectiveness of GABA, benzodiazepines, and barbiturates during withdrawal. More importantly, evidence suggests that the sites of action of benzodiazepines, barbiturates, and neuroactive steroids are differentially modulated by the development of alcohol dependence.

Since allopregnanolone is a potent modulator of GABA_A_ receptors, and because its levels in brain and plasma can reach physiologically active concentrations (see review by Paul and Purdy 1992), the possibility exists that alterations in the sensitivity or synthesis of this steroid with anticonvulsant properties might modulate alcohol withdrawal severity. For example, following 24 hours of alcohol vapor inhalation, plasma allopregnanolone decreased significantly (approximately 40 percent) in WSP mice but not in WSR mice (Finn et al. 1994). Separate studies did not find any change in plasma allopregnanolone in alcohol-dependent male rats following an alcohol-containing liquid diet (Devaud et al. 1996). However, in a group of nine alcohol-dependent human subjects, plasma allopregnanolone and tetrahydrodeoxycorticosterone were significantly lower during early withdrawal than in nonalcoholic subjects (Romeo et al. 1996). Consistent with the hypothesis that steroidal GABA_A_ agonists may modulate withdrawal severity, the decrease in neuroactive steroids during alcohol withdrawal was associated with increased levels of anxiety and depression in the alcoholic subjects.

Chronic alcohol exposure also significantly increases plasma corticosterone levels in WSP mice, with a marginal increase in WSR mice. Corticosterone, a steroid hormone produced by the adrenal glands, plays a role in the body’s response to stress and can increase sensitivity to the development of convulsions in some genotypes. Handling to induce HIC increases corticosterone in WSP mice compared with WSR mice. The magnitude of this increase in corticosterone is greater between handled WSP and WSR mice than between unhandled alcohol-exposed WSP and WSR mice (Finn et al. 1994). Therefore, the increase in plasma corticosterone and decrease in allopregnanolone following chronic alcohol exposure in WSP compared with WSR mice suggests that biological synthesis of these two steroids may be regulated differently in the selected lines. More important, the decrease in plasma allopregnanolone noted previously was observed only in WSP mice and not in a genetically heterogeneous stock of rats. This is consistent with the hypothesis that genetic differences in alcohol withdrawal severity are modulated by both convulsant (i.e., corticosterone) and anticonvulsant (i.e., allopregnanolone) steroids, which have dramatically different mechanisms of action.

### NMDA Receptor Complex

Alterations in excitatory amino acid receptors have been reported following chronic exposure to alcohol (see reviews by Crews et al. 1996; Tabakoff and Hoffman 1996). Most important in this regard is the *N*-methyl-d-aspartate (NMDA) receptor, which responds to the excitatory amino acid glutamate. The NMDA receptor, which regulates an ion channel permeable to calcium and sodium, plays a role in memory, learning, and the generation of seizures.

Researchers have investigated the function of the NMDA receptor-ion channel complex using MK-801, an NMDA antagonist thought to bind within the ion channel. Chronic alcohol administration in a liquid diet produced a 16-percent increase in the number of MK-801 binding sites in the hippocampus, a brain region involved in memory processes and convulsions. Chronic alcohol administration also increased the number of glutamate binding sites. Although these alterations have not been found consistently, evidence suggests that chronic alcohol administration may increase the number of NMDA receptor complexes in the hippocampus.

The time course for the increase in MK-801 binding sites correlates with the onset of withdrawal: The increase in binding sites occurs at the onset of withdrawal and at peak withdrawal (i.e., by 8 hours), but dissipates when symptoms of withdrawal are no longer apparent (i.e., after 24 hours). Additional support for the involvement of NMDA receptors in alcohol withdrawal is provided by the demonstration that alcohol withdrawal can be reduced by administration of MK-801, whereas withdrawal convulsions are exacerbated by doses of NMDA that are not convulsant in control animals.

Results of studies on withdrawal severity in WSP and WSR mice are inconclusive. Initially, research suggested that WSP mice never exposed to alcohol (i.e., alcohol-naive) had a significantly greater number of MK-801 binding sites in the hippocampus compared with alcohol-naive WSR mice. Chronic alcohol administration in a liquid diet for 1 week significantly increased the number of binding sites in both lines, and the binding sites remained significantly higher in the WSP mice. However, further research disclosed that 24 hours of alcohol vapor inhalation, while producing clear signs of withdrawal in WSP mice, had no effect on MK-801 binding. These differences may result from the different methods of alcohol administration employed.

The observed alterations in the number and function of NMDA receptors following chronic alcohol exposure may result from alcohol-induced changes in the quantity of specific NMDA receptor subunit proteins, which have been reported to occur following chronic alcohol administration. Although the exact molecular processes involved are not fully understood, many studies support the hypothesis that chronic alcohol administration results in supersensitive NMDA receptors.

### Voltage-Sensitive Calcium Channels

Calcium ions are essential to many nervous system functions. Voltage-sensitive calcium channels (VSCC’s) admit calcium into the neuron in response to changes in the electrical properties of the neuron’s outer membrane. Scientists investigating VSCC’s often employ calcium channel antagonists, especially those known as dihydropyridines (DHP’s). Because of their effects on the cardiovascular system, calcium channel antagonists such as the DHP nifedipine (e.g., Adalat^®^) are commonly prescribed to treat high blood pressure and some chronic heart conditions.

Experiments with DHP calcium channel antagonists have provided evidence for the involvement of VSCC’s in withdrawal hyperexcitability (reviewed in Little 1991). For example, chronic alcohol administration increases the number of DHP binding sites by approximately 50 percent. This treatment can produce varying degrees of mild withdrawal without spontaneous convulsions. Of interest, the number of binding sites increased after 3 to 4 days of chronic alcohol exposure and was modulated by calcium flow into neurons (see Little 1991).

Consistent with these results, genetic regulation of neuronal calcium channels also occurred after chronic alcohol administration. After exposure to alcohol vapor for 72 hours, the WSP mice experienced a significantly greater compensatory increase in DHP binding sites in brain and heart muscle tissue than did the WSR mice.

Additional studies found that the administration of DHP calcium channel antagonists reduced all forms of alcohol withdrawal hyperexcitability in slices of hippocampus removed from experimental animals immediately after cessation of chronic alcohol administration. In addition, administration of the DHP’s nitrendipine and nimodipine reduced spontaneous and handling-induced alcohol withdrawal convulsions at doses that had little sedative action. Furthermore, administration of a DHP during chronic alcohol consumption prevented the upregulation of DHP binding sites in the brain and significantly reduced withdrawal severity as measured by tremor and convulsions. This decrease in alcohol withdrawal hyperexcitability in animals given nitrendipine plus alcohol was also demonstrated in hippocampal slice preparations. In conclusion, both behavioral and biochemical evidence suggest that increases in DHP-sensitive binding sites may underlie some adaptations to alcohol at the neuronal level, which, in turn, can produce behavioral adaptations and influence withdrawal severity. Additional clinical research is needed to confirm this hypothesis.

## Conclusions

Because chronic alcohol administration alters various neurochemical systems, it is unlikely that one specific mechanism induces physical dependence. More important, the time course for changes in receptor function and sensitivity (Sanna et al. 1993; Watson and Little 1995) indicate that the pattern of neuronal changes occurring during alcohol withdrawal is complex. Currently, researchers do not know whether changes in a particular neurochemical system contribute to one specific symptom of alcohol withdrawal or whether alteration in one neurochemical system generates a cascade of neurochemical changes that manifest as the withdrawal syndrome.

Genetic animal models will continue to be useful in tracing the pathways from complex drug responses to specific genes. QTL mapping and new molecular biological techniques, coupled with the homology between mouse and human chromosomal maps, will facilitate the identification and study of specific genes relevant to the alcohol withdrawal syndrome in humans. In addition, the QTL findings in mice can be compared with the results obtained through the Collaborative Study on the Genetics of Alcoholism (COGA), which is a large-scale, multidisciplinary research program to investigate the genetic components of the susceptibility to alcohol abuse and dependence.

Overall, physical dependence on alcohol produces abnormalities in a number of neurotransmitter systems, resulting in reduced inhibitory function and increased activity of excitatory systems during alcohol withdrawal. By understanding the relative importance and contribution of these neurochemical changes to the alcohol withdrawal syndrome, researchers can develop more effective treatment strategies in humans to minimize increased withdrawal severity in subsequent episodes.

## Figures and Tables

**Figure 1:**
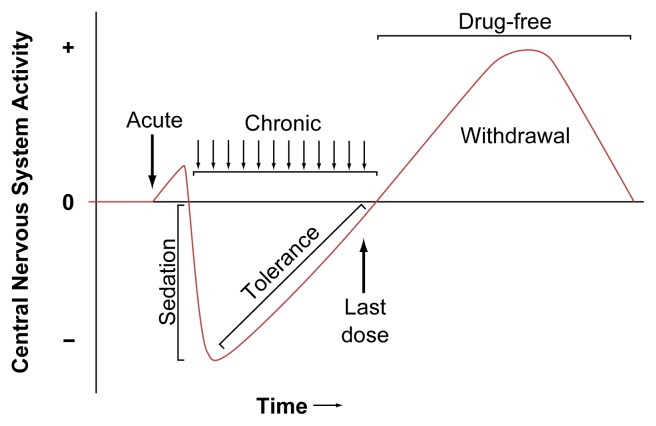
Alcohol’s effect during exposure and withdrawal. The zero line represents a hypothetical measure of overall brain excitability. For most physiological or behavioral measures, acute alcohol administration produces short-term stimulation (i.e., points above the zero line), followed by depression or sedation (i.e., points below the zero line). Continued alcohol administration causes neuronal adaptations that reduce alcohol’s initial perturbing effects and result in tolerance. Physical dependence indicates that alcohol is needed to balance the neuronal adaptations and maintain normal brain function. Removal of alcohol from the body induces a rebound stimulatory effect, resulting in hyperexcitability of the nervous system (i.e., withdrawal syndrome). SOURCE: Adapted from Metten and Crabbe 1996.

## Footnotes

1 For a definition of this and other technical terms used in this article, see central glossary, pp. 177–179.

2 The GABA_B_ receptor, which is another GABA receptor subtype, has a different mode of action and is therefore not discussed here.
